# Expression Patterns of GmAP2/EREB-Like Transcription Factors Involved in Soybean Responses to Water Deficit

**DOI:** 10.1371/journal.pone.0062294

**Published:** 2013-05-07

**Authors:** Juliana Marcolino-Gomes, Fabiana Aparecida Rodrigues, Maria Cristina Neves Oliveira, Jose Renato Bouças Farias, Norman Neumaier, Ricardo Vilela Abdelnoor, Francismar Corrêa Marcelino-Guimarães, Alexandre Lima Nepomuceno

**Affiliations:** 1 Department of Biology, State University of Londrina, Londrina, Brazil; 2 Brazilian Enterprise for Agricultural Research–Embrapa Soybean, Londrina, Brazil; 3 Embrapa LABEX US Plant Biotechnology at ARS/USDA Plant Gene Expression Center, Albany, New York, United States of America; Karlsruhe Institute of Technology, Germany

## Abstract

Soybean farming has faced several losses in productivity due to drought events in the last few decades. However, plants have molecular mechanisms to prevent and protect against water deficit injuries, and transcription factors play an important role in triggering different defense mechanisms. Understanding the expression patterns of transcription factors in response to water deficit and to environmental diurnal changes is very important for unveiling water deficit stress tolerance mechanisms. Here, we analyzed the expression patterns of ten APETALA2/Ethylene Responsive Element Binding-like (AP2/EREB-like) transcription factors in two soybean genotypes (BR16: drought-sensitive; and Embrapa 48: drought-tolerant). According to phylogenetic and domain analyses, these genes can be included in the DREB and ERF subfamilies. We also analyzed a *GmDRIP*-like gene that encodes a DREB negative regulator. We detected the up-regulation of 9 *GmAP2/EREB*-like genes and identified transcriptional differences that were dependent on the levels of the stress applied and the tissue type analyzed (the expression of the *GmDREB1F*-like gene, for example, was four times higher in roots than in leaves). The *GmDRIP-like* gene was not induced by water deficit in BR16 during the longest periods of stress, but was significantly induced in Embrapa 48; this suggests a possible genetic/molecular difference between the responses of these cultivars to water deficit stress. Additionally, RNAseq gene expression analysis over a 24-h time course indicates that the expression patterns of several *GmDREB*-like genes are subject to oscillation over the course of the day, indicating a possible circadian regulation.

## Introduction

Soybeans (*Glycine max* L. Merrill) are one of the most important cultivated oil crops due to their use in human and animal feed and their potential as a biofuel. Despite the increasing improvements in productivity that have been obtained in the last few years, soybean production shows significant losses during drought events. Classified as “sensitive to drought,” especially during the emergence period, soybean crop productivity decreases drastically under water deficit conditions, which may be amplified by the impacts of global warming in the near future [1].

Different mechanisms are used by plants to protect themselves against water deficit; these include changes in stomatal conductance mediated by the hormone abscisic acid (ABA) [2], osmotic adjustment [3], the accumulation of osmoprotectant molecules in the cytosol, which protects cell structures [4], and the activity of antioxidant proteins [5]. The interaction between different physiological mechanisms, triggered by the up- and down-regulation of many genes, demonstrates that water deficit tolerance is a multigenic process. Precise control of this complex network of metabolic pathways allows plants to tolerate periods of water deficit. In this context, transcription factors (TFs) play an important role in this process, from stress-signal perception to transmission via signal transduction pathways and the triggering of different defense mechanisms.

Molecular responses to water deficit can be divided into ABA-dependent and ABA-independent pathways [6]. In the ABA-independent pathway, transcription factors from the AP2/EREBP (APETALA2/Ethylene Responsive Element Binding Protein) superfamily, also known as AP2/ERF (APETALA2/Ethylene Responsive Factor), activate the *cis*-elements that are present in the promoters of stress-induced genes [7]. The AP2/EREBP superfamily is composed of the AP2, ERF, and RAV families. The ERF family includes the ERF and CBF/DREB subfamilies, which are involved in plant responses to abiotic stress, such as water deficit [6], [8].

Of the CBF/DREB subfamilies, the most well-studied transcription factors are DREB1 and DREB2 [9]. The CBF/DREB transcription factors have an ERF domain, which consists of 58–60 amino acids that recognize and bind to GCC-box and C-repeat CRT/Dehydration Responsive Element (DRE) motifs in the target genes [10]. In spite of these common domains, At*DREB1* has been implicated in cold stress responses [11], whereas the functions of the At*DREB2* genes have been mainly described in response to water deficit and osmotic stress [12], [13]. Using an *in silico* analysis strategy, Wang and colleagues [14] searched for *Arabidopsis thaliana* genes with DRE motifs and identified 474 target genes to which the DREB transcription factors might bind. Of these genes, 160 were responsive to abiotic stresses, 27 of which were specifically regulated in response to water deficit. In addition, another genome-wide analysis of DREB-like transcription factors led to the identification of 36 *CBF/DREB*-like genes in *Vitis vinifera* [15], 57 genes in *Arabidopsis thaliana,* 52 genes in *Oryza sativa* [16], 77 genes in *Populos trichocarpa* [17], and 36 genes in *Glycine max* [18].

Overexpression of the *DREB* genes in many crop plants increased abiotic stress tolerance to high temperatures, low temperatures, salt stress, and water deficit [12], [19], [20]. However, studies have shown that the DRIP (DREB-Interacting Protein) [21] and PIF7 (Phytochrome-Interacting Factor 7) [22] proteins negatively regulate the *DREB* genes. Although the exact mechanisms of activation of the DREB transcription factors remain unclear, there is significant evidence indicating that the stability of the DREB proteins in the nucleus plays an important role in their activation [22]. The DRIP proteins contain a C3HC4 RING domain and can act as E3 ubiquitin ligases that mediate DREB ubiquitination and degradation. This indicates that DRIP proteins are important negative regulators of the DREB transcription factors and, therefore, of the DREB-mediated responses to abiotic stresses.

We have previously conducted studies on water deficit responses in soybean focusing on the tolerance that is conferred by the overexpression of the DREB transcription factors. In one of these studies, a drought-sensitive soybean cultivar, BR16, was transformed with the *AtDREB1A* gene to generate the novel soybean line P58. These genetically modified plants showed enhanced water deficit tolerance compared with the wild [23]. Therefore, it is important to identify and characterize the expression patterns of soybean orthologs/paralogs of the *DREB* genes, which could be used to improve water deficit tolerance through genetic engineering approaches similar to those used for *A. thaliana* genes. Using quantitative PCR, we characterized the expression of ten differentially expressed genes from the AP2/EREB and DRIP family identified using subtractive libraries constructed from two contrasting soybean genotypes (BR 16 and Embrapa 48) subjected to water deficit. We also evaluated the influence of the time of day in the expression patterns of these genes using RNAseq to analyze gene expression over the course of the day.

## Materials and Methods

### 1. Plant Materials and Experimental Design

#### 1.1. For subtractive library and qPCR assays

The experiments were performed as described by Rodrigues et al. [24]. Briefly, leaves and roots from control and stressed plants were obtained from Embrapa 48 and BR 16 soybean cultivars that had been grown hydroponically, as described by Martins et al. [25]. When the plants reached the V4 stage, they were subjected to progressive water deficit treatments. Leaves and roots from both cultivars were harvested after 25, 50, 75, 100, 125, and 150 min of exposure to dehydration conditions. To construct the subtractive libraries, the samples were grouped, and the L1 (25 and 50 min of dehydration), L2 (75 and 100 min of dehydration), and L3 (125 and 150 min of dehydration) leaf samples and the corresponding R1, R2, and R3 root samples were formed. The differentially expressed transcripts that were obtained were sequenced by Next Generation Sequencing, and the data were deposited in the Genosoja Soybean Database (http://lge.ibi.unicamp.br/soybean) [26], which was created by a Brazilian Consortium for the Soybean Genome (Genosoja project) and used to search for differentially expressed genes. For the qPCR analysis, we used the same experimental design, but each period of exposure to dehydration (25, 50, 75, 100, 125, and 150 min) was evaluated individually.

#### 1.2. For RNAseq assays

The seeds from the BR16 genotype were cultivated in peat pots (Jiffy) with Supersoil® (Scotts Miracle-Gro Company, Marysville, Ohio, USA). The plants were grown in growth chambers set to 14 h light/10 h night cycles, with 500 µmol m-2s-1 of white light provided by cool white fluorescent bulbs. The temperatures in the growth chamber were set to 28°C during the light period and 20°C during the dark period. Fifteen days after germination, when the plants reached the V2 developmental stage (according to Fehr and colleagues [27]), water was withheld in the stress treatments to induce a water deficit. The soil moisture was calculated by the gravimetric humidity (GH), which corresponds to the percentage of water in the soil in relation to the dry weight of the soil. The volume of irrigation was adjusted to 70% (GH) (near field capacity) for the unstressed treatment, 30% GH for the water deficit stress treatment. Fully expanded V1 leaves were collected from the six plants in each treatment at 4-h intervals from the time the lights came on and were immediately frozen in liquid N2 and stored at −80°C until further use. The samples obtained in the dark were collected with the aid of a small green LED light (PhotonLight.com).

### 2. Gene Identification, Domain Analysis, and Phylogeny

Using the Genosoja Soybean database, 11 target genes that were up-regulated by water deficit were selected based on their similarity to genes from the AP2/EREBP superfamily and the DRIP proteins. Phylogenetic relationships between the *AP2/EREB-like* genes and the *AP2/EREB* genes from Fabaceae were considered. For this purpose, the amino-acid-deduced sequences of the genes were subjected to global alignment, and a phylogenetic tree was constructed with the ClustalW tool of the Molecular Evolutionary Genetics Analysis version 5.0 (MEGA 5) software package [28] using the Neighbor-Joining (NJ) method with the following parameters: Poisson correction, pairwise deletion, and bootstrap (1000 replicates; random seed). Given that the AP2 domain is important for the function and classification of transcription factors from the AP2/EREBP subfamilies, the AP2 domains of the proteins encoded by the selected genes were identified by screening using the ScanProsite online tool (http://prosite.expasy.org/scanprosite/). The amino acid sequences were also aligned using MEGA 5 software [28] through the ClustalW algorithm to assess the pattern of conservation and the differences between the sequences.

### 3. Gene Expression Analysis by qPCR

#### 3.1. Primer design and efficiency analysis

Primers for the target genes were designed based on the GeneModels of the selected genes using the program Primer Express 3.0 (Applied Biosystems/Life Technologies, Grand Island, NY, USA) (Table S1). Primer sequences were determined for the 3' end of each gene, and the amplicons spanned up to 150 base pairs (bp). Primer sequences were BLASTed against the soybean genome (Phytozome database v1.0, http://www.phytozome.net/search.php) to verify the specificity of each primer, and standard curves were produced from serial dilutions of a cDNA pool to estimate the efficiency of the PCR amplification reactions. The primer concentrations were adjusted to obtain efficiency rates higher than 85%, as detailed in Table S1.

#### 3.2. Selection of endogenous genes

To measure relative gene expression, it is essential to normalize the raw data using endogenous genes. However, endogenous gene expression can vary depending on the experimental treatment or time point or the plant developmental stage [29]. The endogenous genes β-tubulin (Glyma20g27280) [30], α-tubulin (Glyma08g12140) [30], Elongation factor 1-β (Glyma13g04050) [30], β-actin (Glyma15g05570) [31], rRNA 18S (Glyma13g12030) [31], and Glyceraldehyde-3-phosphate dehydrogenase (GAPDH) (Glyma06g01850) [31] (Table S1) were assayed to determine the most stably expressed gene in the water deficit-stressed soybean plants. The expression stability of the endogenous genes was evaluated in the leaves and roots of the drought-sensitive BR16 cultivar [32] and was measured using the GeNorm [33] and NormFinder [34] programs. Ct values were transformed into relative quantities using standard curves, and the data were transformed according to the Δ-Ct formula described by Vandesompele et al. [33]. The most stable endogenous genes were chosen.

#### 3.3. Expression analysis

elative expression levels of the target genes *GmAP2/EREB*-like and *GmDRIP*-like were measured in root and leaf samples from Embrapa 48 and BR16 plants. For each time point (0, 25, 50, 75, 100, 125, and 150 min under water deficit), three biological replicates, each with three technical replicates, were analyzed. After DNAse treatment (Invitrogen/Life Technologies, Grand Island, NY, USA), high quality total RNA was used to synthesize cDNA strands (Superscript II First Strand Synthesis, Invitrogen/Life Technologies, Grand Island, NY, USA), and cDNA quality was verified using a standard PCR reaction with an actin primer that spanned an intronic region. After carrying out the amplification efficiency analysis, the genes were amplified by qPCR using a 7500 RT-qPCR Thermocycler (Applied Biosystems/Life Technologies, Grand Island, NY, USA) with the following cycling parameters: 50°C for 2 min, 95°C for 10 min, 45 cycles at 95°C for 2 min, 60°C for 30 seconds and 72°C for 30 seconds. Data were collected during the extension phase, and dissociation curves were performed by heating each amplicon from 60 to 95°C and taking readings at one-degree intervals to verify the specificity of the primers.

The Rest2009 software package [35] was used to evaluate the data because this program provided a more robust statistical analysis. The normalization of the real-time quantitative RT-PCR was performed by taking the geometric average of the selected endogenous genes (Elongation factor 1-β and β-actin), and the control plants (0 min under stress) were used to normalize the relative expression. Hypothesis testing was used to determine whether the differences between the control and treatment conditions were significant [35].

### 4. Gene Expression Analysis by RNAseq

The soybean transcriptome was analyzed in leaf samples from BR16 plants. After DNase treatment (Life TechnologiesGrand Island, NY, USA), high-quality total RNA was used to analyze the transcripts for each time point: 8, 12, 16, 20, 24, and 4 h. Bulks of leaves from two plants were used in the RNA extraction to compose one replication. Three replications for each time point/treatment were sequenced. The RNAseq libraries were built using the Nugen-Ovation® kit according to the manufacturer’s instructions (NuGEN Technologies Inc., San Carlos, CA, USA). The libraries obtained were subjected to sequencing by Illumina HiSeq2000 (Illumina, San Diego, CA, USA). Mapping of the reads was performed with the Soybean genome (Phytosome Glycine max v1.1) using the GeneSifter platform (http://www.geospiza.com/Products/AnalysisEdition.shtml). To compare gene expression between different times and conditions, we log_2_-transformed the normalized reads per mapped million (RPM) value. We then ran a variance test (t-test for two group comparisons or ANOVA equal variance for multiple group comparisons).

## Results

### 1. Gene Identification and Analysis of Domains and Phylogeny

**Figure 1:**
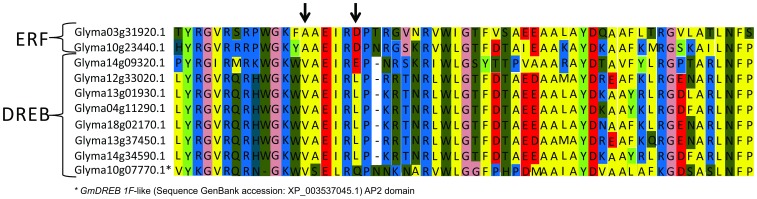
Amino-acid sequence alignment of the AP2 domains. Regions of amino-acid conservation are shown. Letters represent the amino acids of the protein sequences, and dashes delimit the specific 14^th^ and 19^th^ positions for each DREB or ERF subfamily member.

After evaluating differentially expressed genes in soybean plants that had been subjected to short-term water deficit, we selected ten genes with similarity to the AP2/EREB family. We also found one gene encoding a GmDRIP-like protein that, although a negative regulator of DREB genes in *A. thaliana*, was induced in the same water deficit conditions in soybean. The identification of the selected genes, their BlastX similarities and their ontologies are shown in Table 1. By analyzing the AP2 domain of the proteins using the ClustalW algorithm (MEGA 5), we classified ten genes as members of the ERF and DREB subfamilies (Fig. 1). Because the Glyma10g07770.1 annotation in the Phytozome v1.0 database lacked the AP2 domain, we used the predicted protein sequence of X*GmDREB 1F*-like gene (Sequence GenBank accession: XP_003537045.1), which was very similar to Glyma10g07770.1 (Table 1) and contained an AP2 domain.

**Table 1:** Selected DRIP and ERF superfamily target genes.

	BLASTx NCBI			BLAST Gene Ontology	
Gene Model	Description	e-value	GenBankaccession	Biological process	Molecular function
Glyma03g31920.1	Uncharacterized proteinLOC100818907[Glycine max]	2.00E-148	ACU20075.1	GO:0006355: regulation of transcription,DNA-dependent,	GO:0003700: sequence-specificDNA-binding transcriptionfactor activity
Glyma10g23440.1	PREDICTED: ethylene-responsive transcriptionfactor ERF105-like	4.00E-91	XP_003535939	GO:0006355: regulation of transcription,DNA-dependent	GO:0003700: sequence-specificDNA-binding transcriptionfactor activity
Glyma10g07770.1	PREDICTED: dehydration-responsive element-bindingprotein 1F-like [Glycine max]	1.00E-106	XP_003537045.1	GO:0045893: positive regulation of transcription,DNA-dependent, GO:0009409: response to cold,GO:0009414: response to water deprivation	GO:0003700: sequence-specificDNA-binding transcriptionfactor activity
Glyma14g09320.1	Dehydration responsiveelement binding proteinDREB1 [Glycine max]	1.00E-124	AAP47161.1	GO:0045893: positive regulation of transcription,DNA-dependent, GO:0009409: response to cold,GO:0009414: response to water deprivation	GO:0003700: sequence-specificDNA-binding transcriptionfactor activity
Glyma18g02170.1	PREDICTED: ethylene-responsive transcriptionfactor ERF060-like	3.00E-180	XP_003552083.1	GO:0009873: ethylene mediated signaling pathway,GO:0006355: regulation of transcription, DNA-dependent, GO:0009409: response to cold,GO:0009416: response to light stimulus,GO:0006970: response to osmotic stress,GO:0009414: response to water deprivation	GO:0003700: sequence-specificDNA-binding transcriptionfactor activity, O:0005515:protein-binding
Glyma04g11290.1	Dehydration responsiveelement-binding protein 3[Glycine max]	1.00E-136	AAZ03388.1	GO:0009873: ethylene mediated signaling pathway,GO:0006355: regulation of transcription, DNA-dependent, GO:0009409: response to cold,GO:0009416: response to light stimulus,GO:0006970: response to osmotic stress,GO:0009414: response to water deprivation	GO:0003700: sequence-specificDNA-binding transcriptionfactor activity, O:0005515:protein-binding
Glyma13g01930.1	Dehydration responsiveelement-binding protein 3[Glycine max]	6.00E-65	AAZ03388.1	GO:0006355: regulation of transcription, DNA-dependent, GO:0009409: response to cold,GO:0006970: response to osmotic stress,GO:0009414: response to water deprivation,	GO:0003700: sequence-specificDNA-binding transcriptionfactor activity, GO:0005515:protein-binding
Glyma14g34590.1	Dehydration responsiveelement-binding protein 3[Glycine max]	1.00E-66	AAZ03388.1	GO:0006355: regulation of transcription, DNA-dependent, GO:0009409: response to cold,GO:0006970: response to osmotic stress,GO:0009414: response to water deprivation,	GO:0003700: sequence-specificDNA-binding transcriptionfactor activity, GO:0005515:protein-binding
Glyma12g33020.1	Drought responsiveelement binding protein 5[Glycine max]	2.00E-178	CCF23313.1	GO:0006355: regulation of transcription, DNA-dependent, GO:0009409: response to cold,GO:0006970: response to osmotic stress,GO:0009414: response to water deprivation,	GO:0003700: sequence-specificDNA-binding transcriptionfactor activity
Glyma13g37450.1	Drought responsiveelement binding protein 5[Glycine max]	7.00E-103	CBZ41765.1	GO:0006355: regulation of transcription, DNA-dependent, GO:0009409: response to cold,GO:0006970: response to osmotic stress,GO:0009414: response to water deprivation,	GO:0003700: sequence-specificDNA-binding transcriptionfactor activity
Glyma19g34440.1	PREDICTED: E3 ubiquitinprotein ligase DRIP2-like[Glycine max]	0.0	XP_003553481.1	GO:0016567: protein ubiquitination, GO:0009414:response to water deprivation,	GO:0005515: protein binding,GO:0004842: ubiquitin-proteinligase activity

The BLAST description and Gene Ontology are presented for each gene, and the sequences with greater similarity were used (GenBank access #). The BLAST results are from Aug. 2012 and the GO terms for Biological Process and Molecular Function are listed in the Gene Ontology annotation.

The BLAST description and Gene Ontology are presented for each gene; the sequences with greater similarity were used (GenBank accession #). The BLAST results are from Aug. 2012, and the GO terms for Biological Processes and Molecular Function are listed in the Gene Ontology annotation.

**Figure 2:**
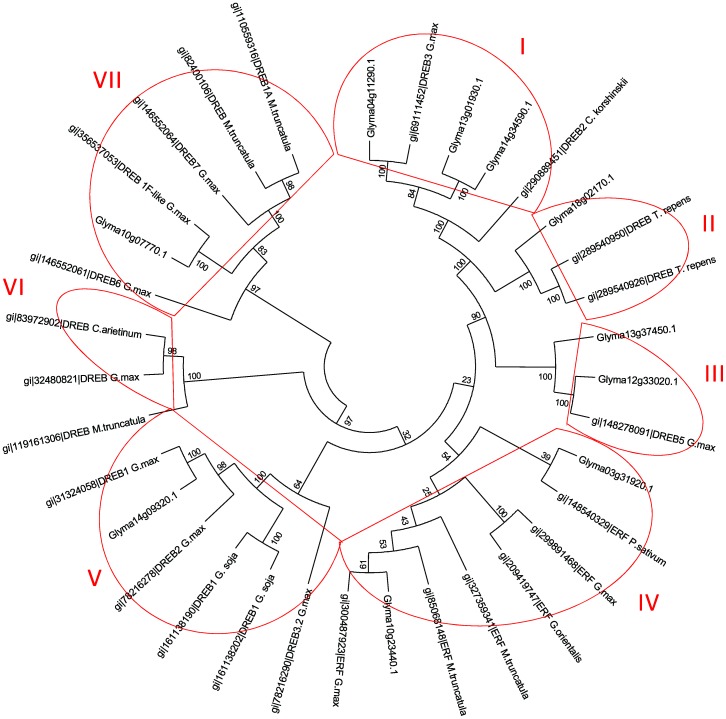
Phylogenetic tree. Proteins encoded by the candidate genes and the DREB/ERF protein that was described in the NCBI database were used to construct the tree using the ClustalW algorithm with the MEGA 5 program. The Neighbor-Joining (NJ) method was used with the following parameters: Poisson correction, pairwise deletion, and bootstrap (1000 replicates; random seed). Candidate genes are represented by the GeneModels, and the homologous DREB/ERF sequences from Fabaceae (*Glycine max, Medicago truncatula, Cypripedium arietinum, Trifolium repens, Glycine soja, Caragana korshinskii, Pisum sativum,* and *Galega orientalis*) are represented by GI.

The translated amino-acid sequences for these genes were also globally aligned, and a phylogenetic tree was constructed. This analysis shows the phylogenetic relationships between the soybean genes from the DREB and ERF subfamilies and other plants of the Fabaceae family that are found in the NCBI database (Fig. 2). This analysis identified seven main groups of genes: (I) soybean DREB3 and *Caragana korshinskii* DREB2; (II) *Trifolium repens* DREB; (III) soybean DREB5; (IV) ERF subfamily; (V) soybean *DREB1* and *DREB2*; (VI) *C. Arietinum*, soybean, and *M. truncatula* DREB; (VII) soybean *DREB6, DREB7* and *DREB 1F* and two *M. truncatula DREB*.

### 2. Analysis of Endogenous Genes

**Figure 3:**
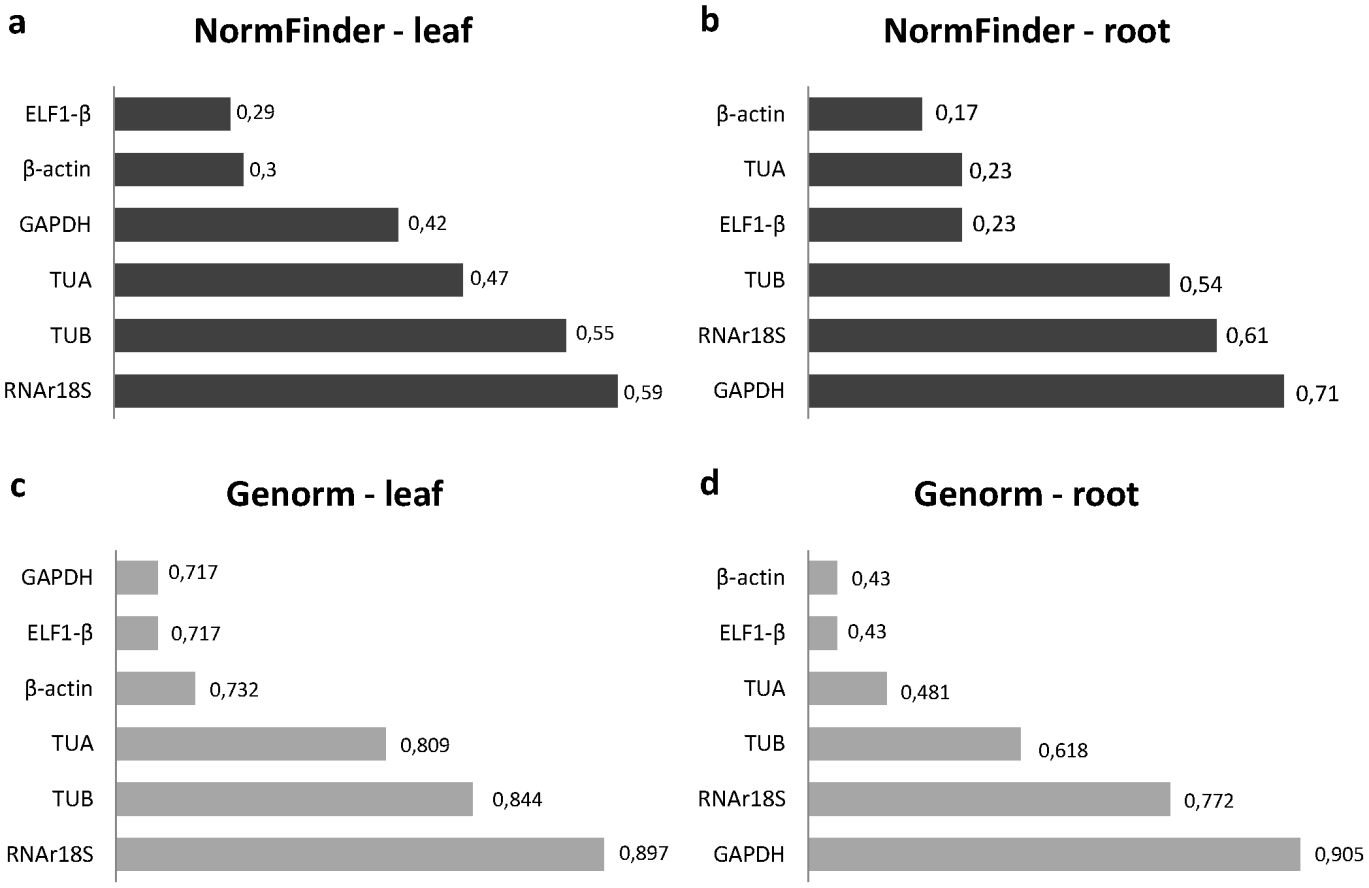
Stability analyses of endogenous genes. In total, six candidate genes were evaluated using the NormFinder and GeNorm programs to select the most stable genes. The *Y* axis represents the *Expression Stability Measure (M)* from the GeNorm program and the Stability value from the NormFinder program. Genes are ranked from less stable (higher values) to most stable (genes with lower values).

Based on results obtained using the NormFinder software package, the Elongation Factor 1β (ELF-1β) and β-actin genes were the most stably expressed in the leaf samples, whereas in the roots, the α-tubulin, ELF-1β and β-actin genes were also significantly stable (Figs. 3A and B).

Based on the analysis performed using the GeNorm program, we found that the ELF-1β and β-actin genes were also the most stably expressed in the roots (Fig. 3D). However, in the leaves, the gene expression of ELF-1β and GAPDH was most stable (Fig. 3C), and β-actin was classified as being the third-most stably expressed gene (Fig. 3C).

### 3. Relative Expression of the *AP2/EREB* and *DRIP*-like Genes in Response to Drought

**Figure 4:**
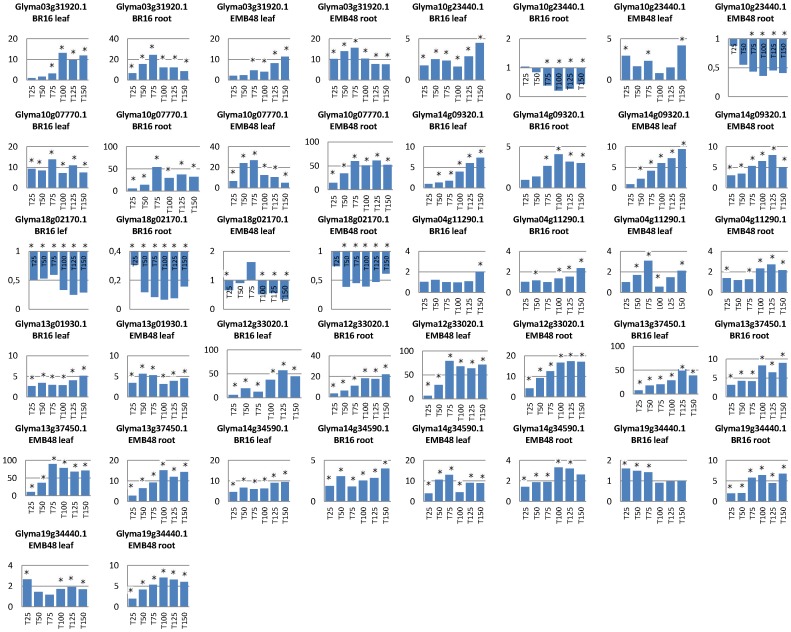
Quantitative PCR of the *AP2/EREB* genes. Gene expression was measured in root and leaf tissues of BR 16 and Embrapa 48 soybean cultivars that were subjected to different periods of water deficit (25 to 150 min). The raw data were normalized to the expression of the *ELF1-β* and the *β-actin* endogenous genes, and the relative expression was determined and compared with the control sample (T0 min).

We evaluated the expression patterns of ten of the *GmAP2/EREB*-like genes and detected nine genes that were up-regulated when both Embrapa 48 and BR16 soybean plants were exposed to water-deficit conditions (Fig. 4). We also detected the up-regulation of a *GmDRIP*-like gene in both cultivars in response to water deficit. The Rest2009 software package allowed for the determination of statistical significance, as detailed in Table S2. The genes showed different transcriptional patterns throughout the water-deficit treatment (25 to 150 min under dehydration) and within the analyzed tissues. Of the up-regulated genes, the *GmDREB1F*-like gene (Glyma10g07770.1) and the GmDREB5-like genes (Glyma12g33020.1 and Glyma13g37450.1) showed the highest stress-induced expression in both cultivars (Fig. 4). In contrast, Glyma18g02170.1 was repressed or non-differentially expressed in response to water deficit during the evaluated stress periods.

### 4. Influence of Time of the Day on GmDREB-like Genes Responses to Water Deficit

**Figure 5:**
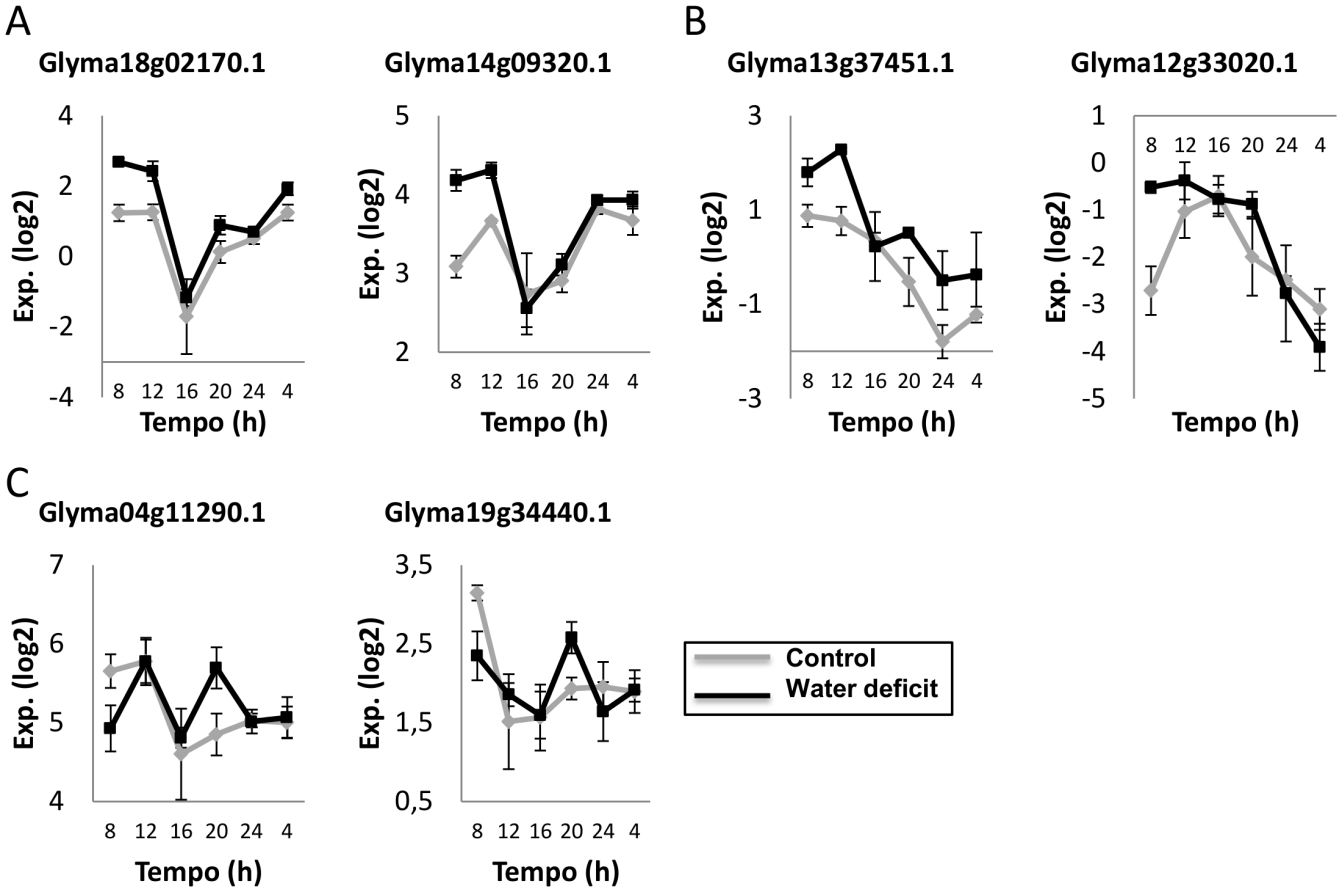
RNAseq data of the *AP2/EREB* and *DRIP2-*like genes. Gene expression was measured in leaf tissues of BR 16 soybeans subjected to water deficit (drought) and normal hydration conditions (control). Gene expression (Exp. Log. [log_2_]) was evaluated over a 24-h time course from the time the light came on (ZT0) in 4-h intervals. Error bars represent the standard error (SEM).

To observe if the target genes oscillated in their expression patterns over the course of the day, we used the RNAseq reads from the BR16 genotype, which were mapped in the soybean genome. Under control conditions, the expression of the DREB-related genes generally increased just before dawn and reached their peak expression between 8 and 12 h (Fig. 5). Interestingly, the stress induction of these genes also displayed a strong oscillation over the course of the day. Based on the gene expression patterns in response to water deficit, the genes we analyzed could be divided into three groups: group A was characterized by high expression levels during the end of the night and the beginning of the day, with lowest expression levels in the afternoon (16 h); group B corresponded to genes with higher expression levels in the beginning of the day and low expression levels at the end of the day; group C was represented by genes significantly induced by water deficit stress approximately 2 h before lights went out, at 20 h.

## Discussion

### 1. Identification of Target Genes

The large AP2/EREB family includes transcription factors that share one or two domains (AP2/EREB) and function in plant development and physiological processes that are related to abiotic stress responses [36]. The DRIP transcription factors act as negative regulators of the AP2/EREB family in *A. thaliana*, and these proteins specifically regulate the DREB subfamily. Therefore, studies aiming to evaluate the transcriptional profiles of genes from these families are important for understanding the relationship between the antagonistic transcription factors AP2/EREB and DRIP.

Bioinformatics has been a powerful tool for *in silico* analyses because databases have grown rapidly over the last several years due to the large amount of data that has been generated by genomics and transcriptomics techniques. The gene expression data that was stored in the soybean database [24] was useful for identifying *AP2/EREB*- and *DRIP-*family transcripts that were differentially expressed under water-deficit conditions. The differentially expressed transcripts were present in the soybean genome in the Phytozome database v1.0 (http://www.phytozome.net/search.php), which allowed us to examine correlations between the expressed transcripts and the soybean gene models (Glymas). Gene anchorage provided information about the size of the sequence, coding sequence regions, and the 3' and 5' untranslated regions (UTRs), which was essential for phylogenetic analyses and primer design. The gene models are putative genes that are predicted through *in silico* analyses of the soybean genome. Our study provides experimental proof of the functionality of these genes and describes the predicted genes as *in vivo*-expressed genes. Additionally, the BlastX tool [37] has allowed for the identification of probable proteins that are coded by those transcripts and their attributed ontologies through searches in the GenBank [http://www.ncbi.nlm.nih.gov/genbank/] and Gene Ontology [http://www.geneontology.org/] databases (Table 1). Importantly, the majority of the genes that were evaluated in our study were similar to predicted and uncharacterized proteins, showing, once again, the importance of this study to the description of these genes as expressed genes.

Amino acids that are located at the 14^th^ and 19^th^ positions of their protein sequence are highly conserved in AP2/EREB subfamily members. The transcription factors from the DREB subfamily contain a conserved valine (V14) and glutamic acid (E19) at these positions, whereas the ERF subfamily has a conserved alanine and aspartic acid at these positions [38]. Of the *AP2/EREB*-selected target genes, two belong to the ERF subfamily, and seven belong to the DREB subfamily (Fig. 1). However, of the DREB members, we noticed a higher degree of conservation at the 14^th^ position than at the 19^th^ position. This subfamily classification was confirmed by a phylogenetic analysis, which showed the separation of the DREB and ERF subgroups into distinct segments (Fig. 2). We also observed the formation of some smaller groups within the DREB and ERF subfamilies that were related to soybean and other Fabaceae genes (Fig. 2). The close phylogenetic relationship between the target genes and genes from the AP2/EREB family corroborates the similarity that was found by BLASTing the sequences; this can indicate homology of function for sequences that present high phylogenetic relatedness, as suggested by Oh et al. [39].

### 2. Endogenous Genes

The endogenous stability analysis performed using the GeNorm and NormFinder programs generally indicated that ELF-1β and β-actin were the most stable endogenous genes (Fig. 3). However, in leaf tissues, the GeNorm program identified the GAPDH gene as one of the two most stable endogenous genes instead of β-actin (Fig. 3C and D). However, the M value (expression stability measure) for the *GAPDH* gene differed by only 0.015 units from that of the β-actin gene (the third-best endogenous gene) (Fig. 3C), and the *GAPDH* gene showed a low stability in evaluations of root tissues (Fig. 3A and C). Based on these results, we selected the *ELF-1β* and β-actin endogenous genes as expression controls for the qPCR analysis.

### 3. Transcriptional Profiles of the Target Genes

Studies have previously been conducted to evaluate the expression of the *DREB* and *ERF* soybean genes in response to stresses such as cold and water deficit [13], [18], [40]. However, the genes that were evaluated in our study were predicted to be candidate genes in soybean via *in silico* analysis. To our knowledge, our work using subtractive libraries and qPCR analysis of expression are the first experimental reports of the expression of these genes in response to water deficit in soybeans. We have quantified the expressions of the genes after different periods of time under stress – which correspond to different levels of stress – in both root and leaf tissues. The results provide an abundant set of information regarding the expression of these genes in response to water deficit in the BR16 and Embrapa 48 soybean cultivars.

We observed an increase in the expression of the *GmDREB1*-like gene (Glyma14g09320.1) in leaf tissues that was proportional to the increase in the severity of the stress (from 25 min to 150 min of dehydration) (Fig. 4). In addition to the differences in expression over time, we also observed differences in the gene expression profiles between the root and leaf tissues. For example, the *GmERF*-like gene (Glyma10g23440.1) was only induced in response to water deficit in the leaves, whereas, in the root tissues, its expression was repressed (Fig. 4). This gene was previously identified in forwarded subtractive libraries as being expressed in the leaf tissues of BR 16 plants [24], which corroborated the findings of this study. This contrasting behavior in different tissues should be highlighted because it might indicate that the promoter acts in a tissue-specific manner. Candidate genes with tissue-specific promoters are interesting due to their potential for use in biotechnology applications [41].

The *GmDREB5*-like genes (Glyma12g33020.1 and Glyma13g37450.1) and the *GmDREB1F*-like gene (Glyma10g07770.1) also showed differential expression patterns in root and leaf tissues (Fig. 4). For example, Glyma10g07770.1 was expressed two-fold higher in the roots than in the leaves of Embrapa 48 plants that had been exposed to 75 min of water deficit, and this gene was expressed more than four-fold more in the roots than in the leaves of the BR16 cultivar at the same time point (75 min). This gene (Glyma10g07770.1) is highly similar to a predicted soybean *DREB1F*-like sequence (Table 1) and has a close phylogenetic relationship to the DREB subfamily (Fig. 2). However, it was not possible to identify an AP2 domain, which is typical of this type of gene, in the Glyma10g07770.1 sequence. Our global alignment analysis shows that the Glyma10g07770.1 sequence is very similar to the *in silico*-predicted soybean DREB1F-like sequence that was deposited at the NCBI, but the Glyma protein lacks the initial 52 amino acids that encode a major portion of the AP2 domain, likely due an incorrect annotation of the protein sequence in Phytozome v1.0. Based on our data, we propose, for the first time, the inclusion of Glyma10g07770.1 in the AP2/EREB family and report that this gene is water deficit-inducible. In contrast, the expression of the *GmDREB5*-like genes (Glyma12g33020.1 and Glyma13g37450.1) was higher in the leaves than in the roots of both cultivars (Fig. 4), which indicates differences between the responses of each tissue to stress. This finding is consistent with those of Wang et al. [42], who reported increased expression of a DREB subfamily gene (*CkDBF*) in the leaves of *C. korshinskii* after 4 h of dehydration, whereas the expression level of this gene in the roots was only moderate [42]. These genes are similar to the DREB5 sequence in soybean and comprise group III of the phylogenetic tree that was presented in Fig. 2. The *GmDREB5* gene was identified in soybean; however, currently, there is no published information on the expression of the sequence that is deposited at NCBI (GenBank: ABQ53928.1).

The Glyma04g11290.1, Glyma13g01930.1, and Glyma14g34590.1 genes were similar to the soybean *DREB3* gene (Table 1) and were placed in phylogenetic Group I (Fig. 2), which justifies the denomination of these genes as *GmDREB3*-like genes. The *GmDREB3* gene was recently identified in the soybean genome, and its expression was associated with plant responses to cold [40]. Although it was first identified in cold stress responses, the authors reported that superexpression of the *GmDREB3* gene in transgenic plants increased their tolerance to water deficit; however, gene expression analyses in non-transgenic plants were unable to confirm the responsiveness of the *GmDREB3* gene to water deficit. Our results show that *GmDREB3*-like genes (*Glyma04g11290.1, Glyma13g01930.1* and *Glyma14g34590.1*) are up-regulated in response to water deficit in the BR 16 and Embrapa 48 genotypes (Fig. 4). Additionally, the global alignment and phylogenetic analyses highlight the close correlation between the *GmDREB3*-like genes and DREB2 of *Caragana korshinskii* (*CkDBF*), which comprise group I of the phylogenetic tree (Fig. 2). *C. korshinskii* is a plant that is adapted to areas with limited water availability, and it is typically found in desert areas of China [42]. Superexpression of *CkDREB2* in tobacco triggered various stress-related genes and enhanced the response of transgenic plants to water-deficit stress. Hence, this finding highlights the importance of the orthologous soybean *DREB3*-like genes (*Glyma04g11290.1, Glyma13g01930.1* and *Glyma14g34590.1*) that were studied here.

To understand the molecular mechanisms of the response to water deficit, we also analyzed the expression of a *GmDRIP2-*like gene (*Glyma19g34440.1*), which is differentially expressed in soybean BR16 leaf subtractive libraries and encodes the protein DRIP2-like, which ortholog is a negative regulator of DREB factors in *A. thaliana*. Plant responses to water stress involve several genes that are involved in signaling via a complex metabolic network. Transcription factors play a key role in this signaling network, and plants are able to respond to stress efficiently only when this regulation is precisely controlled. Dong and Liu [43] evaluated the action of the DREB repressor molecule RAP2.1 and concluded that repressors are important for maintaining tight control of the stress responses and preventing metabolic damage and wear caused by a “runaway” stress response [43]. Here, we found that the repressor *GmDRIP2*-like gene was up-regulated at different time-points after the induction of water deficit in the Embrapa 48 and BR 16 cultivars (Fig. 4). Considering that this higher level of mRNA could potentially result in higher levels of the GmDRIP2-like protein, this may be an important mechanism for maintaining tight control of the water deficit response, as has been proposed for other DREB repressors [43]. However, a difference in the gene expression of the *GmDRIP*-like gene was evident in the leaves and roots; specifically, higher expression of this gene was observed in the roots compared with the leaves (Fig. 4) for both cultivars. Additionally, in the roots, expression of this gene increased over the time-course as the severity of the water deficit stress increased. This evidence suggests that different mechanisms control the water deficit response in different soybean tissues. Furthermore, some differences in the expression of this gene were observed between the cultivars. For example, in the BR16 leaves, the *GmDRIP2*-like gene was not induced during the longest exposures to stress (100, 125, and 150 min), whereas in Embrapa 48, an increase in gene expression was observed during these exposures (Fig. 4). Therefore, this variation could be a genetic/molecular difference between the responses of these cultivars to water deficit stress.

### 4. Influence of Time of the Day on the Expression Pattern

According to the gene expression patterns in response to water deficit, the genes identified in the RNAseq analysis were divided into groups A, B, and C. Group A is composed of *Glyma18g02170.1* and *Glyma14g09320.1* genes, which had the highest expression levels during the end of the night and the beginning of the day and the lowest expression levels at 16 h. The *Glyma18g02170.1* gene, as previously mentioned, is phylogenetically related *to DREB3* from *T. repens* (group II), a gene identified through structural genomics studies [44]. Until recently, there has been no public data on the transcriptional patterns of this gene. Despite the induction of *Glyma18g02170.1* could not be detected by qPCR analyses, in the RNAseq analysis this gene was induced and displayed day oscillation in response to water deficit stress, indicating that the circadian clock might influence the water deficit responses in soybeans. The difference in the transcription patterns between the qPCR and RNAseq data can be explained by disparity in plant developmental stages (V4 and V2 for qPCR and RNAseq, respectively), as demonstrated in previous studies in which changes in gene expression were detected between the developmental stages of *Arabidopsis* [45] and soybean [46].

According to the BLAST and Phylogenetic analyses (Table 1 and Fig. 2), the genes *Glyma12g33020.1* and *Glyma13g37450.1* are related to *GmDREB5.* Interestingly, both genes exhibited similar expression patterns in response to water deficit over the course of the day: the highest expression levels at the beginning of the day and the lowest expression levels at the end of the day. These genes comprise group B (Fig. 5). To date, our results are the first showing the influence of the time of the day on *GmDREB5* gene expression. This suggests that the circadian clock seems to be acting to modulate the expression profiles of genes with related functions so as to be coordinately expressed in the best period of the day, contributing to an efficient water deficit response. The circadian control of related genes was previously described in soybean studies in which transcripts encoding proteins with distinct roles in seed metabolism and biochemistry were segregated by phase/time of day [47].

Group C is composed of the genes *Glyma04g11290.1* and *Glyma19g34440.1*. *Glyma04g11290.1* is similar to a *GmDREB3* gene (Fig. 2, Table 1), and *Glyma19g34440.1* encodes a DRIP2-like protein, which, in *Arabidopsis,* regulates the expression of DREB factors. Although the interaction between DREB and DRIP has been investigated at the protein level, there is a lack of information regarding these genes’ relationship at the transcript level. Based on qPCR expression data, we previously proposed that *GmDRIP2*-like expression might be part of an important mechanism for maintaining tight control of the water deficit response. Supporting this idea, our RNAseq data indicates that the *GmDRIP2*-like gene is significantly induced by water deficit coordinately with a soybean *DREB* gene (*DREB3*-like) (Fig. 5).

### Conclusion

Here, we provide an abundant set of information concerning the expression of AP2/EREB transcription factors in different tissues of the Embrapa 48 and BR 16 Brazilian soybean cultivars in response to varying water deficit levels. We detected differences in gene expression that depended on (1) the level of stress that was applied and (2) the tissue that was evaluated, where contrasting behavior within different tissues might indicate promoters that act in a tissue-specific manner.

The genes that were evaluated here are predicted gene models in soybean (identified through *in silico* analyses), and most of the genes were similar to predicted or uncharacterized proteins. To our knowledge, our results using Subtractive libraries and qPCR analyses are the first evidence of the expression of these genes in response to water deficit in soybeans. We also experimentally identified a new *AP2/EREB*-like gene (*Glyma10g07770.1*) and showed its up-regulation by water-deficit stress conditions. Furthermore, we believe that the present study is the first report on the up-regulation of *GmDREB3*-like and *GmDREB5*-like genes in response to water deficit in soybeans. The differential expression patterns of the *GmDRIP*-like gene in the BR16 and Embrapa 48 cultivars is the first reported genetic/molecular difference between these cultivars in response to water deficit.

Additionally, our results show that several *DREB-like* and the *DRIP2-like* genes have daily oscillation patterns in their expression profiles. This suggests a circadian clock control over these genes, even under water deficit conditions. The circadian clock is known to confer adaptive advantages to organisms by synchronizing the best time of the day for biochemical reactions to occur to optimize development or to endure stressful situations. Thus, improving our understanding of the oscillation patterns of important water deficit stress effectors, such as *DREB* genes, becomes of great interest.

## Supporting Information

Table S1 **Primers for the target genes.** Nucleotide sequences and the denaturation temperatures (Tm) of the Forward (F) and Reverse (R) primers are shown. The amplification efficiency was calculated using a standard curve.
(XLSX)

Table S2 **Statistical analysis.** The Rest 2009 software package was used to calculate iterations of gene expression among all experimental treatment periods and their statistical significances. P(H1) is the probability of observing the difference between the control plants and those exposed to water deficit by chance. The *Result* columns indicate the direction of the change in expression when *p*<0.05 (*UP* = up-regulated; *DOWN* = down-regulated; *ND* = not differentially expressed).
(XLSX)
